# Effect of K_2_O/SrO on structural, thermal, optical, and mechanical properties of SiO_2_–B_2_O_3_–SnO_2_ glass for IT/LT-SOFC applications

**DOI:** 10.1039/d5ra10040b

**Published:** 2026-05-18

**Authors:** Manish Kumar, Akshay Kumar, K. Singh

**Affiliations:** a Department of Physics, Sardar Patel University Mandi Himachal Pradesh-175001 India; b Department of Physics and Material Science, Thapar Institute of Engineering and Technology Patiala-147004 Punjab India kusingh@thapar.edu

## Abstract

A new series of borosilicate glasses (40SiO_2_–15B_2_O_3_–(40 − *x*)K_2_O–*x*SrO–5SnO_2_, *x* = 0, 5, 10, 15, 20, 25, 30, 35, 40 mol%) are synthesized *via* a melt-quenching technique. X-ray diffraction of the as-prepared glasses confirmed the amorphous nature and phase separation in all the glasses. The phase separation tendency increases with the addition of SrO in place of K_2_O in the glasses. Structural analysis revealed that with the sequential substitution of the monovalent K^+^ cation by the divalent Sr^2+^ cation, there was a significant effect on the silicate Q^*n*^ and borate BO_3_ structural units. The optical band gap lies in the insulating range from 4.36 to 4.26 eV and decreases with SrO concentration, which makes the glasses good for use as sealants. The hardness was measured using Vicker’s indentation technique, and lies in the range of 5.4 to 7.0 GPa, comparable to other reported glass sealants. The thermal expansion coefficient (TEC) of the *x* = 20 and 25 glasses could be suitable for solid oxide fuel cell (SOFC) applications.

## Abstract 2

SiO_2_–B_2_O_3_–K_2_O–SrO–SnO_2_ glasses were developed and evaluated to check their suitability as sealants for IT/LT-SOFC applications.

## Graphical abstract

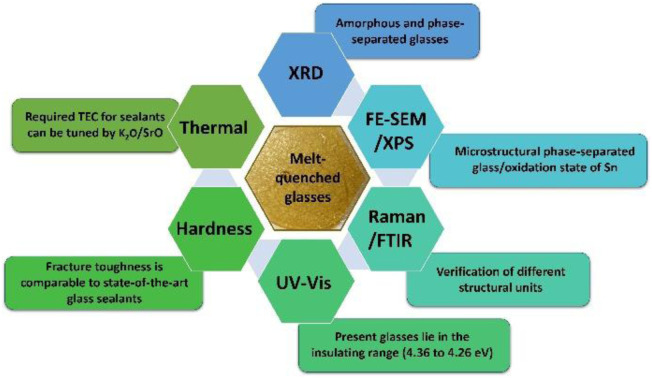

## 1 Introduction

Development of an appropriate glass sealant is a big challenge in commercializing intermediate- or low-temperature solid oxide fuel cells (IT/LT-SOFCs). Extensive research is underway to develop compatible, robust glass sealants for energy-efficient SOFCs for lower-temperature operation (800–500 °C). In most cases, the glass sealants are either silicate-based or borosilicate-based glasses containing alkaline earth metal oxides with variable content along with 10–15 mol% intermediate oxides to optimize the sealing properties.^1–3^ These glass sealants are good for high-temperature SOFCs at >800 °C. To reduce the cost and facilitate commercialization of SOFCs, effort is being applied to develop newer materials (800–500 °C) as sealants for IT/LT-SOFCs. Another significant problem is the variation in the thermal expansion coefficient (TEC) between different components of SOFCs that arises during the high-temperature operation of SOFCs. This can cause mechanical stress, which might result in cracks or leaks. Consequently, each component must have a comparable TEC to develop high-efficiency IT/LT-SOFC devices.^1^

The planar design of SOFCs requires the use of high-temperature sealing materials. This is to avoid any intermixing or fuel leakage among the SOFC’s components for proper functioning.^4–6^ To develop such a sealant, the material needs to be chemically inert (for a longer run), electrically insulating, and have a TEC comparable with other components.^7^ Glasses or glass-ceramics could be a prominent choice for sealant applications due to their compositionally dependent tunable characteristics.^8^ Among them, borosilicate-based glasses are one of the possible choices for glass sealants in SOFCs.^1^ With a few modifications in the composition, the desired properties can be obtained for SOFC applications.^9^ The challenge of simultaneously attaining comparable TECs with other components and long-term stability (thermal or chemical) limits the applicability of glass sealants. To address these problems, research has focused on the fundamentals of glass science and sealing technology.^10,11^ For developing IT/LT-SOFC sealant glasses, the glass transition temperature (*T*_g_) should be lower than the operating temperature of the SOFC. In addition to these, the softening temperature (*T*_s_) must be marginally higher than the operating temperature.^12^

Glasses with an SiO_2_/B_2_O_3_ ratio of around 2 showed a glass transition (*T*_g_) value around 650 °C to 710 °C, and the TEC increases with heat treatment duration.^13^ The BaO/SrO ratio in borosilicate glasses also significantly influences the TEC and other sealing properties. These glasses could be used as a sealant for IT-SOFCs.^14^ It was earlier reported that 40SiO_2_–15B_2_O_3_–27BaO–10MgO–8ZnO exhibits a relatively low *T*_g_ of ∼616 °C with a TEC of ∼10.6 × 10^−6^ K^−1^. The addition of B_2_O_3_ reduces the viscosity and slows crystallization. This will also improve the glass wettability and strengthen its bond with the interconnector/separator (steel).^15^ Additionally, a higher content of B_2_O_3_ lowers the dilatometric softening points while maintaining thermal stability.^16^ Previous studies demonstrated that boron interacts strongly with humidified hydrogen (the fuel used in SOFCs) at working temperatures, resulting in the creation of volatile boron-containing species such as B_2_(OH)_2_ and B_2_(OH)_3_.^17^ As a result, any seal containing a high concentration of boron oxide is prone to degradation under SOFC operating conditions. However, earlier reports also suggest that alkali- and alkaline-earth-containing borosilicate glasses exhibit *T*_g_ ∼ 545 °C to 580 °C and *T*_s_ ∼ 680 °C to 740 °C.^18^

The motive of the present work is to develop glass sealants for IT/LT SOFCs. For this, a borosilicate-based glass series with a SiO_2_/B_2_O_3_ ratio of ∼2.67 was designed to attain the desired results. To optimize the properties required for sealants, two modifiers are used, *i.e.*, K_2_O and SrO. The selection of both modifiers is based on the diagonal relationship between K^+^ and Sr^2+^ in the periodic table, which usually provides some resemblance in their properties. Meanwhile, SnO_2_ is selected to increase the TEC and make the glass more thermally stable, to withstand high temperatures during working conditions.^19,20^ SnO_2_ plays a different role in the glass depending upon the composition, concentration, and degree of Sn oxidation. It can act as a fining agent at higher temperatures, a network modifier at low concentrations, and a network former at higher concentrations.^20,21^ Moreover, SnO_2_ also acts as a stabilizing oxide, strengthening the silicate network, lowering the ion mobility, and improving hydrolytic resistance.^22^ It may prevent or reduce the formation of the B_2_(OH)_2_ and B_2_(OH)_3_ phases under a reducing oxygen atmosphere during SOFC operation. The addition of alkaline earth oxides modifies the thermal properties and also provides resistance to moisture. A moderate addition of SrO provides faster glass formation kinetics. Despite this, at excessively high addition levels, SrO degrades the chemical and thermal properties.^23,24^ Meanwhile, it is well known that K_2_O creates more non-bridging oxygens (NBOs) in the glass network, which reduces the viscosity.^25^ Moreover, if an alkali is added where boron is present, it changes the boron oxide structural units from BO_3_ to BO_4_.^26^ The addition of alkali and alkaline earth oxides breaks the Si–O–Si linkage and creates NBOs. These structural changes significantly influence the properties of glass sealants, as also noted in related glass-based (bio)material systems, wherein network-structure modification led to a change in mechanical properties.^27,28^ So, it is imperative that the structural, optical, mechanical, and thermal properties of proposed glass compositions provide a basis for qualifying as a sealant for IT/LT-SOFCs.

The effects of alkali and alkaline-earth metal oxides on the structural, mechanical, optical, and thermal properties of the borosilicate glasses were investigated to determine their suitability as sealants for IT/LT-SOFCs. X-ray diffraction (XRD), Raman spectroscopy, Fourier-transform infra-red (FTIR) spectroscopy, the Vickers microhardness indenter test, and UV-visible spectroscopy were used to investigate these properties.

## 2 Experimental methods

### 2.1 Glass preparation

Glass samples with the composition 40SiO_2_–15B_2_O_3_–(40 − *x*)K_2_O–*x*SrO–5SnO_2_ (*x* = 0, 5, 10, 15, 20, 25, 30, 35, 40 mol%) were prepared using the melt-quench technique. All precursors used for the sample preparation were of analytical grade: SiO_2_ (Quartz Powder, Loba Chemie), H_3_BO_3_ (99.5%, Merck), K_2_CO_3_ (99.5%, Loba Chemie), SrCO_3_ (98%, Loba Chemie), and SnO_2_ (99.9%, Loba Chemie). The different glasses with their compositions and labels are given in Table 1. A 30 g batch of each sample was mixed using an agate mortar and pestle for 2 hours (h) in a wet (acetone) medium to achieve uniform mixing with a fine particle size of the constituent compounds of the glass composition. The ground powder was placed in a high-temperature resistance furnace for melting at 1550 °C. Intermediate holding steps of the furnace at various temperatures was used to avoid the evaporation of volatile B_2_O_3_ and K_2_O. The melted sample was poured onto a thick copper block and immediately pressed with the help of another thick copper plate to cool it quickly. Quenched samples with a high K_2_O content (up to 25 mol%) and varying SrO (up to 15 mol%) were highly hygroscopic in nature, with poor thermal stability, and were therefore not considered for any further characterization.

**Table 1:** Nominal compositions (mol%) used for developing the borosilicate glasses, along with their sample codes

Sample code	Composition				
	SiO_2_	B_2_O_3_	SnO_2_	K_2_O	SrO
KS-20	40	15	5	20	20
KS-25	40	15	5	15	25
KS-30	40	15	5	10	30
KS-35	40	15	5	5	35
KS-40	40	15	5	0	40

## 3 Characterization

### 3.1 Physical

The physical parameters of the as-prepared glasses are measured and calculated using the standard formulation as given in many research articles.^29,30^ For instance, the density (*ρ*_exp_) of the glass is measured *via* the Archimedes principle using the following equation:1
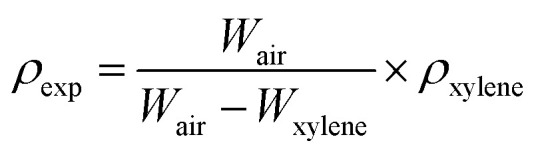
where *ρ*_xylene_ is the density of xylene at room temperature (∼0.863 g cm^−3^) and *W*_air_ and *W*_xylene_ are the weight of the sample in air and xylene, respectively. Additionally, the molar volume (*V*_m_) was calculated by dividing the molecular weight (*W*_m_) of the as-prepared glasses by the measured density. These characteristics, together with compositional data, make it possible to determine other structural parameters, such as ionic concentration (*N*), inter-ionic distance (*r*_i_), oxygen packing density (OPD), field strength (F.S.), and excess molar volume (*V*_e_) using established correlations. These properties provide information on glass network compactness, structural changes, and cation–oxygen interactions within the glass matrix.

### 3.2 X-ray diffraction (XRD)

The nature of the quenched glass samples was confirmed by XRD. The samples were crushed into fine powders with the help of a mortar and pestle. These fine powder samples were subjected to XRD analysis using a Bruker D8 Advance diffractometer (model: Smartlab SE, Rigaku, Japan) equipped with monochromatic Cu-K_α_ (*λ* = 1.54 Å) radiation. The XRD patterns of these pulverized samples were recorded within a range of 10° to 80° with a step size of 0.02°.

### 3.3 Raman spectroscopy

Raman spectroscopy was employed to access information about the vibrational modes and structural mechanism occurring within the sample. Spectra were recorded using a Lab-Ram HR Evolution Raman Spectrometer (Horiba, France) within a range of 10–2000 cm^−1^ equipped with a diode-pumped laser (*λ* = 532 nm) and 1800 lines per mm gratings. To resolve the overlapping bands beyond 1200 cm^−1^, deconvolution was performed using Gaussian curve fitting, which helps to quantify the various bands.

### 3.4 FTIR spectroscopy

To determine the structural information of the glass structure, Fourier-transform infra-red (FTIR) spectroscopy was carried out in attenuated total reflectance (ATR) mode. The diamond ATR accessory allows direct analysis of solid powdered glass samples in the 4000–400 cm^−1^ range and achieves a spectral resolution better than 0.6 cm^−1^, suitable for detailed spectral analysis. For this, a Thermo-Scientific™ Nicolet™ Summit™ X spectrometer was used, which has a scan range of 8000 cm^−1^ to 350 cm^−1^ and features a high signal-to-noise ratio, making FTIR more useful for the precise determination of bands.

### 3.5 Field emission scanning electron microscopy (FE-SEM)

Microstructure and chemical analysis were carried out using a Carl Zeiss Sigma 500 FEG-SEM equipped with energy dispersive spectroscopy (EDS). The FE-SEM images were recorded at an accelerating voltage of 5 kV with a magnification of 5000× and a working distance of around ∼3.0 mm. For chemical point analysis, the images were recorded at an accelerating voltage of 20 kV with a magnification of 5000× and a working distance of around ∼8.1 mm.

### 3.6 X-ray photoelectron spectroscopy (XPS)

The oxidation state of Sn in the prepared glasses was determined *via* XPS. It was performed on polished glass samples using a Thermo Scientific Nexsa G2 Surface Analysis System equipped with a monochromatic Al K_α_ X-ray source (1486.6 eV) with a micro-focused beam, spot size of 200 µm and sensitivity of ∼0.1 atom%. The spectra were collected using a 180° double-focusing hemispherical electron-energy analyzer equipped with a multichannel detector. The peaks were further deconvoluted using a Voigt fitting function and Tougaard background subtraction using the Origin-Lab software.

### 3.7 UV-visible spectroscopy

To determine whether the material was insulating or conducting in nature, UV-Visible spectroscopy was used to determine the optical band gap. The spectrum was recorded on a SHIMADZU UV-2600 UV-visible spectrophotometer. Spectra of the samples in powder form were taken in reflectance mode in the spectral range of 200–800 nm, and the step size was 0.5.

### 3.8 Microhardness

Fracture toughness (*K*_ic_) and brittleness (*B*_i_) are important parameters for SOFC applications. Micro-hardness (*H*_v_) helps to calculate these parameters. Micro-hardness was calculated using the Vickers micro-indentation hardness method on an Omni-Tech instrument. To ensure even load and accurate hardness readings, the sample was well-polished using emery paper with grit numbers 600, 1000, 1200, and 2000. A diamond Vickers indenter on a microhardness testing machine was used to apply a load of 500 grams (4.90 N) with a dwell time of 20 seconds at three different positions on each polished glass surface slice. Through this process, the hardness was determined by measuring the diagonals of developed pyramidal indentations.

### 3.9 Thermal dilatometry study

For developing glass sealants for SOFC applications, the TEC of the sealant material must match the TEC of other components to avoid any thermal shock or stress induced during their operation. To determine the TEC of the samples, a Netzsch DIL 402 PC dilatometer was used. The measurements were taken in the range of 25 to 800 °C in air, with a heating rate of 5 °C min^−1^.

## 4 Results and discussion

### 4.1 Physical properties

**Fig. 1:**
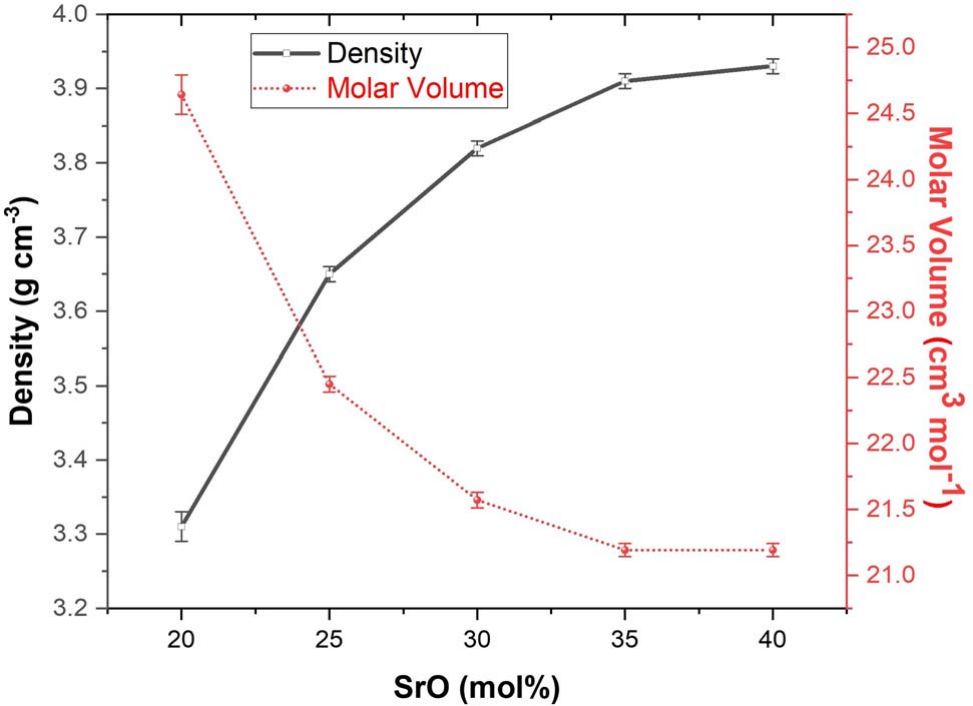
Densities and molar volumes of the as-prepared glasses with variable SrO content in the glasses.

The prepared borosilicate glasses were transparent with a sandy beige color. This is due to the presence of SnO_2_ in the glass composition.^19^ As the concentration of SrO increases, it approaches a light sandy beige color in the glasses. The molar mass of SrO (103.62 g mol^−1^) is higher than that of K_2_O (94.2 g mol^−1^).^31^ This means that when K_2_O is replaced by SrO content, the density must increase in the glasses. The *ρ*_exp_ increased from 3.32 to 3.93 g cm^−3^ as the SrO concentration increased from 20 to 40 mol% in the glasses. The density of strontium-based borosilicate glass is reported to be in the range of 3–4 g cm^−3^.^14^ The molar volume must have the opposite trend to the density according to eqn (2). The densities and molar volumes of the glasses are shown in Fig. 1.

The molar volume and density do not follow a linear relationship with SrO concentration. At higher concentrations (35–40 mol%), the molar volume and density become constant. Similar behavior has been reported for strontium borosilicate glass, in which the SrO content does not affect *V*_m_ within a range of 35–45 mol%.^32^ It is clearly indicated that higher contents of SrO in the present glasses lead to different behaviours than lower contents. The highest depolymerization is observed in the KS-40 glass, which could be associated with the higher field strength of Sr^2+^ than K^+^ cations, creating more NBOs (Table 2).

**Table 2:** Different physical parameter values of the prepared borosilicate glasses, obtained from the density

Glass code	*W*_m_ (g mol^−1^)	*ρ*_exp_ (g cm^−3^)	*V*_m_ (cm^3^ mol^−1^)	Ionic conc. (×10^21^) (ions per cm^3^)		Inter-ionic distance (×10^−8^) (Å)		OPD (mol cm^−3^)	*V*_e_ (cm^3^ mol^−1^)	F.S. (×10^15^) (cm^−2^)	
				*N*_K_	*N*_Sr_	*R*_K_	*R*_Sr_			*F*_K_	*F*_Sr_
KS-20	81.57	3.31	24.64	4.89	9.77	5.89	4.68	71	−2.18	1.77	5.63
KS-25	81.94	3.65	22.45	4.02	13.4	6.29	4.20	78	−3.45	1.56	6.95
KS-30	82.39	3.82	21.57	2.79	16.7	7.10	3.91	81	−3.42	1.22	8.06
KS-35	82.84	3.91	21.19	1.42	19.9	8.89	3.69	82	−2.90	0.77	9.04
KS-40	83.29	3.93	21.19	—	22.7	—	3.53	82	−1.19	—	9.88

Using experimental density and glass composition, other physical parameters are also calculated. The ionic concentration of Sr^2+^ ions (*N*_Sr_) increases with a decrease in the inter-ionic distance of Sr^2+^ ions (*R*_K_). On the other hand, the ionic concentration of K^+^ ions (*N*_K_) decreases with an increase in the inter-ionic distance of K^+^ ions (*R*_K_). The OPD increased with a rise in SrO content, which indicates a tight arrangement of oxygen atoms in the present glasses. On the other hand, F.S. also increased with a rise in SrO, due to the double charge of Sr^2+^ compared to K^+^. The KS-40 sample has a higher OPD and F.S., *i.e.*, 82 and 9.88, respectively. The *V*_e_ is also calculated and given in Table 2. The *V*_e_ decreases up to KS-30; further, it increases with SrO content in the KS-35 and KS-40 glasses, which is also reflected in the density and molar volume of the glasses.

### 4.2 XRD analysis

**Fig. 2:**
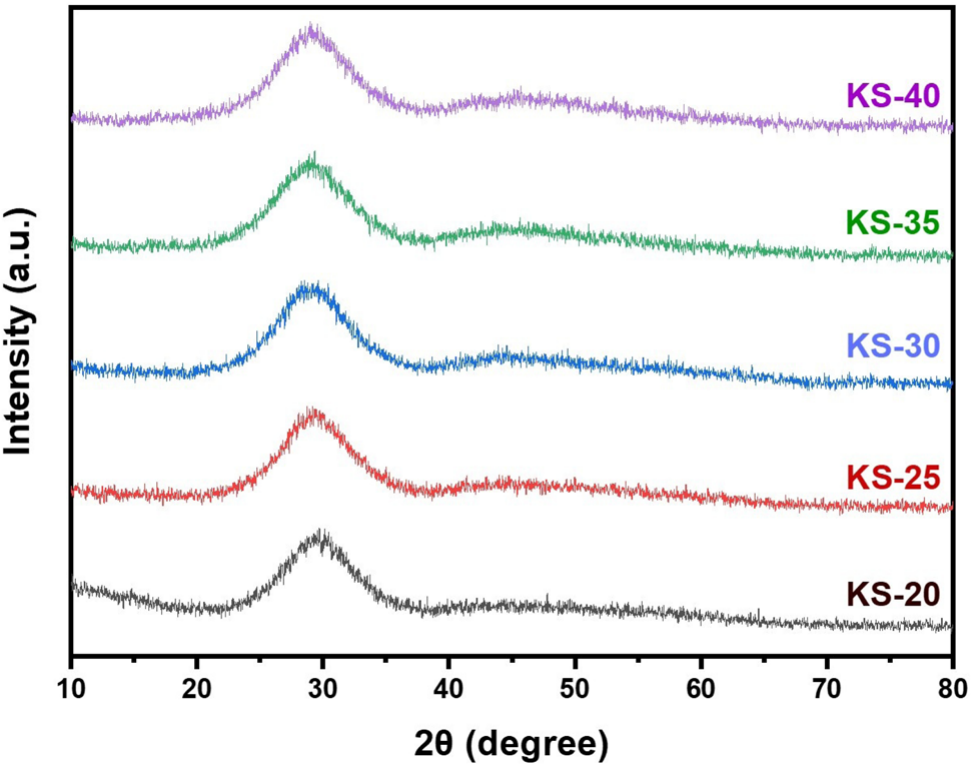
XRD patterns of as-prepared glasses with their sample codes.

The XRD patterns of the prepared glasses (Fig. 2) show a broad hump confirming the amorphous nature of the glasses. This hump is observed at a diffraction angle of ∼20° to 40°. This hump slightly shifts toward a lower diffraction angle with the rise in SrO content from 20 to 40 mol%. Additionally, a very weak second hump is also observed at 2*θ* ranging from 38° to 65°. This hump also shifts toward a lower angle and becomes slightly more prominent with the addition of SrO in place of K_2_O in the glasses. The second hump may be related to phase separation, which was likely caused by the presence of two network formers in these glasses. In glasses, using cations with higher field strengths as network modifiers can result in greater immiscibility and phase separation than using cations with lower field strengths.^33,34^ This can be clearly observed in the XRD patterns of these glasses, where the addition of SrO in the glass increases the tendency for phase separation.

### 4.3 Phase separation

**Fig. 3:**
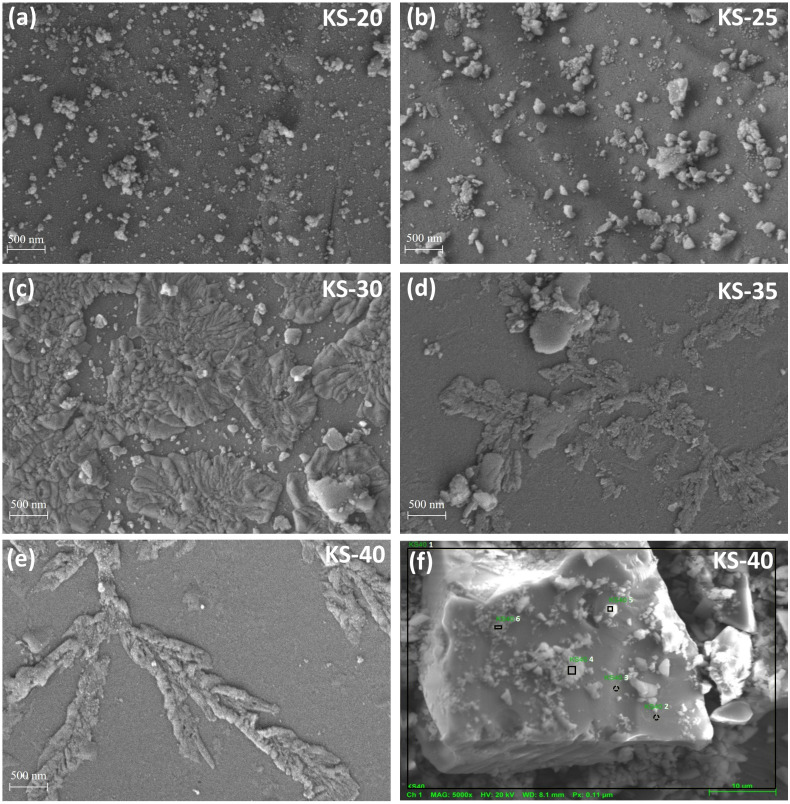
(a–e) FE-SEM micrograph of KS-20–KS-40 glass samples showing the surface morphology within the glass matrix, and (f) point analysis of the KS-40 sample at different positions.

FE-SEM and EDS were employed to confirm the phase separation in the glasses and the elemental distribution across different places in the as-prepared KS-40 glass, respectively. As illustrated in Fig. 3(f), the results indicate a non-uniform distribution of elements across the sample, suggesting the presence of immiscibility-induced phase separation in the glass network (Table 3).

**Table 3:** Point EDS analysis of the KS-40 glass sample at different positions (atom%)

Elements	1	2	3	4	5	6
O	64.26	47.20	40.05	41.24	76.78	64.46
Si	13.97	16.91	15.14	17.13	9.18	14.58
Sr	18.16	22.78	19.98	22.15	12.46	18.65
Sn	2.00	2.41	1.56	2.59	1.58	2.31
B	1.61	10.70	23.28	16.89	0.00	0.00

### 4.4 XPS analysis

**Fig. 4:**
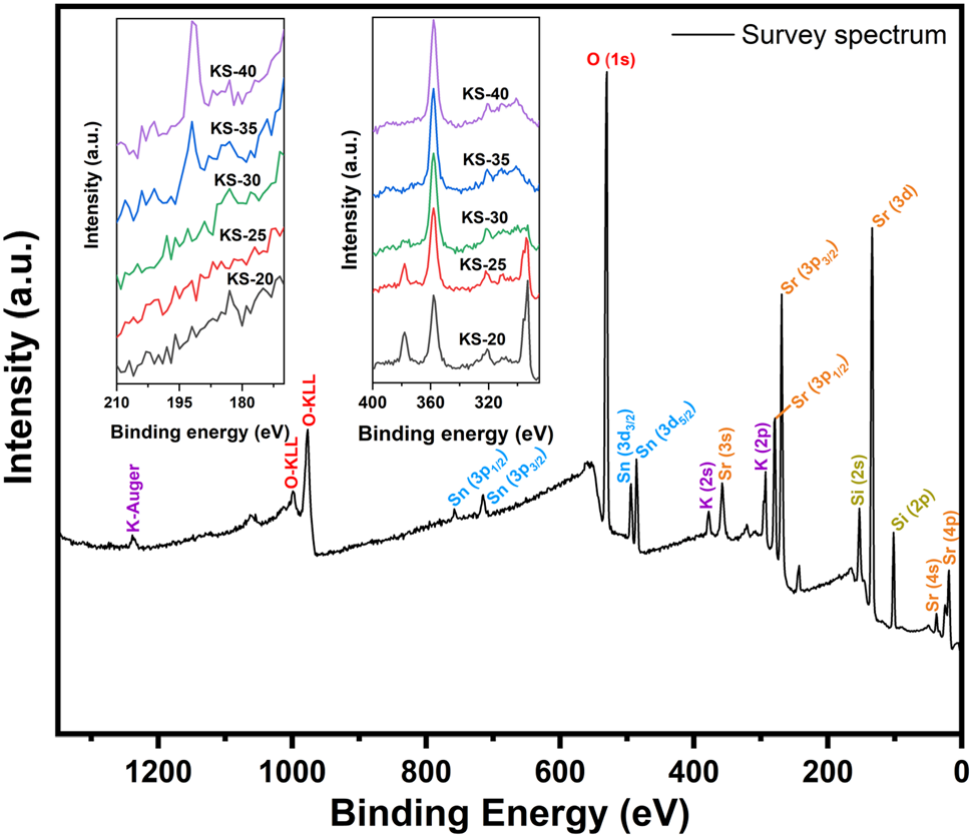
Representative XPS survey spectrum of the KS-20 glass with labelled elemental transitions. The inset highlights the B 1s peak in the 180–196 eV binding energy region and the sequential reduction in K 2p peak intensity from KS-20 to KS-40, consistent with the decrease in potassium concentration across the series.

**Fig. 5:**
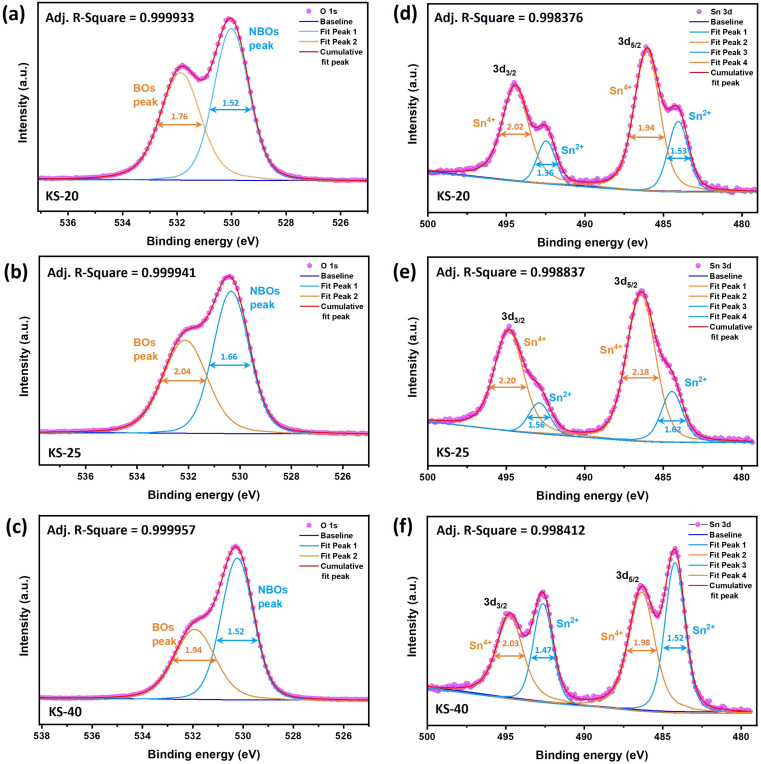
Representative deconvoluted XPS spectra of (a–c) O 1s spectra showing the deconvolution of BO and NBO peaks and (d–f) Sn 3d spectra showing the deconvolution of the Sn^2+^ and Sn^4+^ oxidation states of KS-20, KS-25, and KS-40 glasses, respectively.

XPS is performed on the as-prepared glasses to confirm the oxidation state of Sn. The survey spectrum confirms the presence of Si, B, K, Sr, Sn, and O, consistent with the composition, as shown in Fig. 4. Additionally, the inset highlights the gradual decrease in the intensity of the K 2p doublet peak as the potassium concentration decreases from the KS-20 to KS-40 glasses, showing successful stoichiometric control during the synthesis process. The Sr 3p_3/2_ and Sr 3p_1/2_ peaks are well resolved at binding energies (BE) of 269 eV and 279 eV with a peak splitting of 10.4 eV. Similarly, the 3d orbital of Sr is deconvoluted to identify the d_5/2_ and d_3/2_ peaks at 133 eV and 134 eV, respectively. Additionally, Sr 4p, 4s, and 3s peaks were found at BE 19 eV, 38 eV, and 358 eV, respectively. This result is in good agreement with the reported literature for Sr^2+^ ions in an oxide environment.^35,36^ The K 2p peak features spin–orbit coupling and is composed of two peaks: 2p_3/2_ at ∼293 eV and 2p_1/2_ at ∼295 eV with a splitting of ∼2.77 eV for the KS-20 sample.^37^Fig. 5 shows the representative deconvoluted XPS spectra of KS-20, KS-25, and KS-30 glasses in the O 1s and Sn 3d regions. The O 1s spectrum is deconvoluted to distinguish the overlapped peaks, as shown in Fig. 5(a). The higher-binding-energy peak is attributed to bridging oxygens (BOs), which might correspond to oxygen atoms linking two Si atoms (Si–O–Si). Meanwhile, the lower binding energy peak is attributed to NBOs, which are associated with oxygen bonded to modifier cations and silicon atoms.^38^ One report suggested that peaks at ∼532 and ∼530 eV could be assigned to BOs and NBOs, respectively.^37^ These results are similar to those of the present glasses, where peaks at 530 eV (BOs) and 532 eV (NBOs) indicate that the concentration of NBOs increased with the increase in SrO concentration. However, the peaks at ∼977 eV and ∼999 eV are due to the presence of O-KLL, as reported in the literature.^36^ The B 1s peak lies in the range of 180–196 eV.^39^ The peaks associated with binding energies of ∼102 eV and ∼153 eV correspond to Si 2p and Si 2s, respectively.^40^

The peaks observed at ∼715 and ∼758 eV are attributed to the Sn 3p_3/2_, and 3p_1/2_ peak, respectively.^41^ Another two broad peaks are observed at ∼484 eV and ∼492 eV due to the presence of Sn 3d_5/2_ and Sn 3d_3/2_, respectively, with a peak splitting of ∼8 eV.^42^ The high-resolution spectra of Sn 3d confirmed the presence of Sn^2+^ and Sn^4+^ mixed oxidation states in all glass compositions, as shown in Fig. 5(d–f). Through deconvolution, quantitative analysis found that the +2 oxidation state increases with increasing SrO content, except in the KS-20 glass due to the presence of an equal proportion of alkali and alkaline-earth components. This suggests that at lower concentrations of SrO, the Sn^4+^ oxidation state is dominant due to higher oxygen availability and stronger network connectivity. At higher SrO concentrations, the modifier role of SrO becomes more dominant, leading to depolymerization of the glass network and increased formation of NBOs. This led to more conversion of Sn^4+^ to the Sn^2+^ oxidation state. Thus, the results indicate that SrO concentration significantly influenced the Sn state. The oxidation state and O 1s distributions of these glasses are shown in Table 4.

**Table 4:** XPS-derived analysis showing the distributions of (BOs), (NBOs) and Sn^4+^/Sn^2+^ oxidation states in the prepared glasses

Sample code	O 1s		Oxidation state	
	BOs (%)	NBOs (%)	Sn^4+^ (%)	Sn^2+^ (%)
KS-20	46.29	53.71	75.48	24.52
KS-25	45.63	54.37	81.02	18.98
KS-30	41.85	58.15	71.40	28.60
KS-35	43.42	56.58	65.27	34.73
KS-40	39.84	60.16	53.81	46.18

### 4.5 Raman spectral analysis

**Fig. 6:**
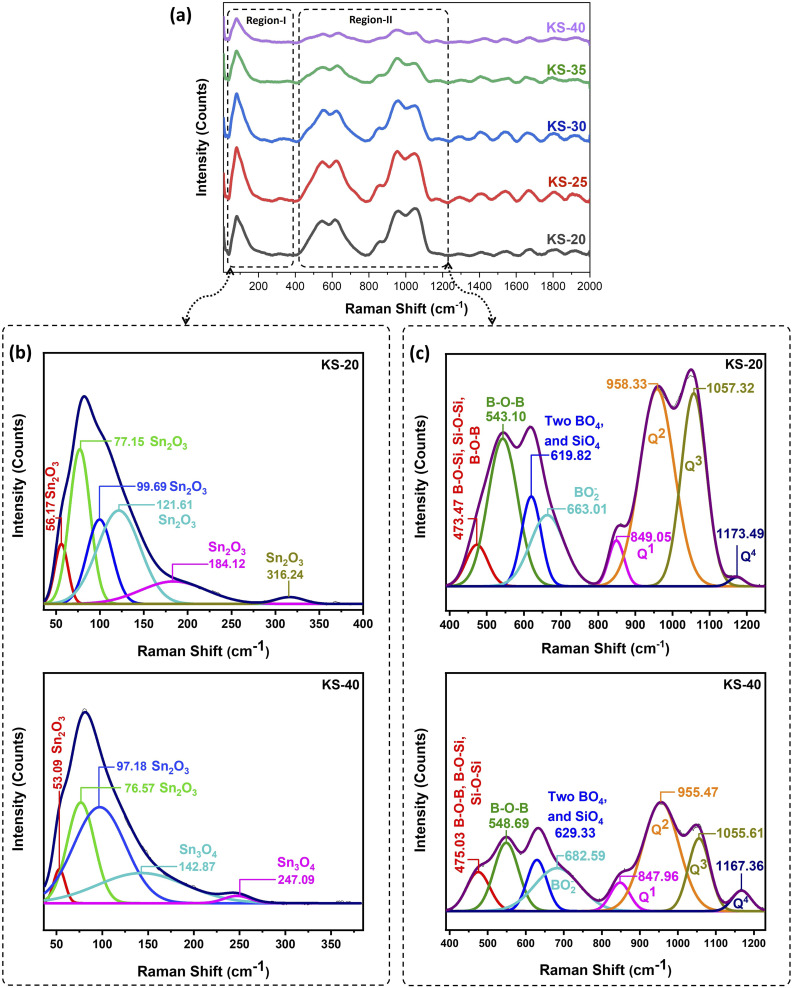
(a) Raman spectra for the prepared borosilicate glass series, (b) representative deconvoluted spectra of KS-20 and KS-40 glasses in the spectral range below 400 cm^−1^, and (c) representative deconvoluted spectra of KS-20 and KS-40 glasses in the range of 400–1200 cm^−1^.

The Raman spectra of the as-prepared glasses are given in Fig. 6(a). Usually, silicate glasses contain four major bands in Raman spectra. These bands are as follows: (i) the 400–700 cm^−1^ band consists of mixed stretching and bending modes of Si–O–Si bonding due to delocalized vibration of the SiO_2_;^43^ (ii) the bands at 850 cm^−1^ and 900 cm^−1^ consist of orthosilicate and pyrosilicate units with zero and one BO atoms (Q^0^ and Q^1^), respectively; (iii) another band occurring in the region of 950–1000 cm^−1^ and (iv) one at 1050–1100 cm^−1^ represent the stretching motion of metasilicate units with two and three BO atoms (Q^2^ and Q^3^), respectively.^44^ The region from 400–800 cm^−1^ represents the symmetric breathing vibrations of six-membered rings with BO_3_ and BO_4_ tetrahedra, diborate, pentaborate, and ring-type metaborate groups. The pyroborate, pentaborate, and tetraborate groups are found in the 800–1000 cm^−1^ range.^45^ Previous studies on tin silicate glasses suggest that SnO_2_ predominantly forms the triangular pyramid polyhedron SnO_3_, with a minor fraction of square pyramid SnO_4_ coordination within the silicate network. This minor SnO_4_ coordination diminishes as the SnO_2_ content rises.^46^

The deconvoluted data below the 400 cm^−1^ region is shown in Fig. 6(b). According to previous studies, the perovskite-type complex oxide SrSnO_3_ exhibits four primary vibrational bands: the SrSnO_3_ lattice modes with irreducible representations B_2g_ (113, 114, 147 cm^−1^) and A_g_ (168, 169 cm^−1^); Sn–O–Sn bending (A_g_) at 220 and 222 cm^−1^; O–Sn–O deformations (A_g_) at 255 and 256 cm^−1^; and Sn–O_3_ stretching (A_g_) at 398 and 400 cm^−1^.^47^ However, these Raman bands do not exist in the present glasses. Another report on tin oxide identifies four different phases, mainly SnO_2_, SnO, Sn_2_O_3_, and Sn_3_O_4_.^48^ Out of these, the Raman bands observed in the present glasses are consistent with Sn_2_O_3_ and Sn_3_O_4_ structures, which exhibit +2 and +4 oxidation states.^42^ This result is strongly supported by the XPS analysis of the Sn 3d core level, which confirms the coexistence of mixed Sn^2+^ and Sn^4+^ states. Because of these, the glasses have a distinct sandy beige color.

The Raman spectra from 400 to 800 cm^−1^ contain two broad bands for the prepared borosilicate glass series, as shown in Fig. 6(c). The deconvoluted Raman spectra for the KS-25, KS-30, and KS-35 glasses are given in SI in Fig. S1. From the deconvoluted spectra, minor peak contributions on the edges of the two broad peaks can be clearly observed for the KS-20 glass. These peaks occur in the regions 459–486 cm^−1^ and 663–709 cm^−1^. Moreover, these peaks can be clearly seen without the deconvolution when the SrO concentration increases in the glasses. The deconvoluted peak at 459–486 cm^−1^ associated with bending or rocking vibrations of B–O–B, B–O–Si, and Si–O–Si linkages, which typically occur at 475 cm^−1^, confirmed the interconnectivity between borate and silicate units in the borosilicate glasses.^49^ The peak at 544–554 cm^−1^ was related to the bending vibrations of B–O–B bonds.^50^ Another major peak occurring in the region of 617–632 cm^−1^ was related to vibrations of borosilicate rings. These rings consist of two BO_4_ and two SiO_4_ tetrahedral structural units and are mostly noticeable when the SiO_2_/B_2_O_3_ ratio lies in the range of 1 to ∼2.6. This is consistent with the danburite structure (characteristic of the borosilicate unit), which shows a peak at 614 cm^−1^.^51,52^ The deconvoluted peak at 663–709 cm^−1^ is associated with vibrations of the metaborate group, which typically occur at 675 cm^−1^.^53^

In the higher Raman range from 800–1200 cm^−1^, it was reported that the small bands found for borate glasses at 960 and 1140 cm^−1^ correspond to different diborate groups.^54^ In borosilicate glasses, the stretching vibrations of the SiO_4_ tetrahedra correspond to the peak at 858 cm^−1^, consisting of three NBOs (Q^1^). The peaks occurring at 961 cm^−1^ and 1050 cm^−1^ consist of two NBOS (Q^2^) and one NBO (Q^3^), respectively.^52^ This intense silicon–oxygen peak was dominated by the weak borate peaks that occur at 960 cm^−1^ and 1140 cm^−1^ in borosilicate glasses. The bands associated with the silicon–oxygen network were redistributed during the rise in SrO content from 20 to 40 mol%. It was found that when alkaline earth metals, as a modifier, exceed a certain concentration, they depolymerize the structure and form isolated SiO_4_ units.^55^ Thus, it is evident that the excess Sr^2+^ depolymerized the Si–O–Si bond, which decreases BOs and increases NBOs. Thus, the peak intensity at 1050 cm^−1^, which was related to the vibrations of one NBO atom (Q^3^), decreased. The decrease in the Q^3^ structural unit might be associated with the increase in SrO content, as Sr^2+^ has a higher field strength than K^+^. This is associated with the stronger binding force between alkaline-earth metals and NBOs. This was confirmed by the decrease in inter-atomic distance with the rise in SrO content. As per reports, alkali field strength might influence the equilibrium Q^3^ ↔ Q^2^ + Q^4^ of silicate species in binary silicates. Additionally, an increase in field strength favors the formation of the Q^2^ + Q^4^ at the expense of the Q^3^ unit.^56^ The peak at 1167 to 1181 cm^−1^ consists of fully polymerized units of silicon tetrahedra (Q^4^).^56^ With excessive SrO content, the stronger bonds Si–O (443 kJ mol^−1^) and B–O (498 kJ mol^−1^) were replaced by the weaker Sr–O bond (138 kJ mol^−1^), leading to the formation of Si–O–Sr or B–O–Sr linkages. This might have happened because Sr^2+^ ions can take up positions in the voids of the silicon–oxygen or boron–oxygen glass network. In other words, higher SrO content resulted in overall higher NBOs, weakened glass structure, and a consequent decline in structural stability. This might be the reason for the decrease in Raman intensity for the KS-35 and KS-40 samples. The repetition of a small band was observed at higher frequencies (above 1200 cm^−1^), possibly arising from diffraction.

**Fig. 7:**
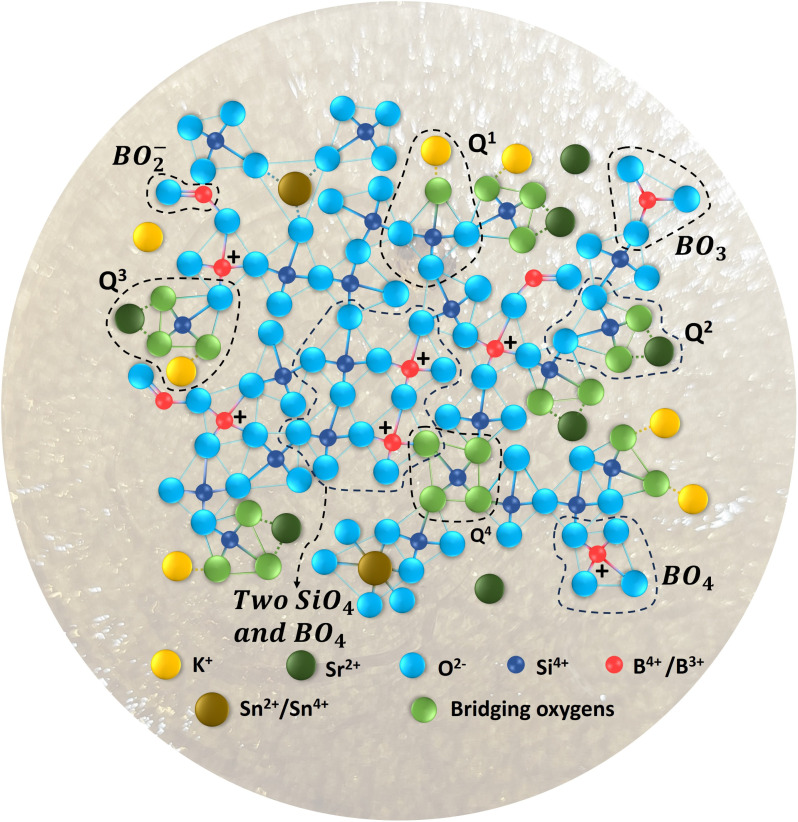
Based on Raman analysis, a structural model of the KS-20 glass network is proposed.

As shown in Table 5, the Q^2^/Q^3^ ratio obtained from the Raman band intensities changes with replacement of K_2_O by SrO. A higher Q^2^/Q^3^ ratio indicates more effective network depolymerization. The increase in Q^2^/Q^3^ from KS-20 to KS-40 shows a gradual breakdown of the silicate network, possibly due to the addition of modifiers (K_2_O and SrO). The impact of this increased disorder can also be seen in the high TEC of these glasses (Section 4.9). Based on the Raman results, the possible glass structures are proposed and given in Fig. 7.

**Table 5:** Silicate band intensity in the 800 to 1200 cm^−1^ region, and the determined the ratio (Q^2^/Q^3^)

Sample code	Band centers (with intensity)				Ratio
	Q^1^	Q^2^	Q^3^	Q^4^	(Q^2^/Q^3^)
KS-20	849 (0.23)	958 (0.93)	1057 (0.92)	1173 (0.06)	1.0
KS-25	849 (0.26)	953 (0.90)	1052 (0.84)	1174 (0.07)	1.1
KS-30	849 (0.24)	954 (0.84)	1053 (0.70)	1182 (0.07)	1.2
KS-35	855 (0.32)	952 (0.78)	1049 (0.65)	1177 (0.21)	1.2
KS-40	847 (0.20)	955 (0.55)	1055 (0.39)	1167 (0.17)	1.4

### 4.6 FTIR analysis

The FTIR spectra at the higher end of the wavenumber region (3780–3600 cm^−1^) exhibit bands consisting of the stretching modes of silanol (Si–OH) groups, as shown in Fig. S2 (SI).^57^ The band around the 3100–2770 cm^−1^ wavenumber region corresponds to hydrogen bonding caused by stretching vibrations of H_2_O in a glassy matrix.^57^ The band occurring around the region of 2350–1650 cm^−1^ mainly consists of hydroxyl stretching vibrations in a glassy matrix.^58^

**Fig. 8:**
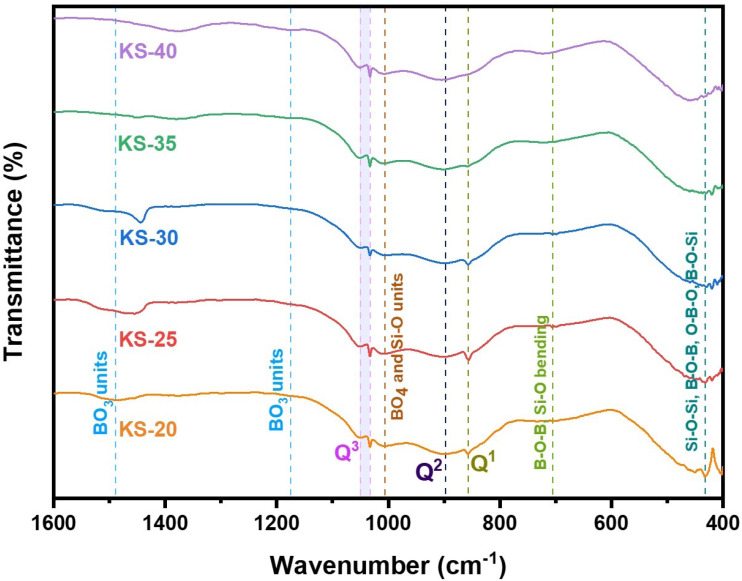
Enlarged fingerprint region of the FTIR spectra (1600–400 cm^−1^) highlighting Si–O–Si and B–O vibrational bands.

The wavenumber range of 1600–400 cm^−1^ mostly corresponds to silicate and borate units (Fig. 8). The band at 1600 to 1300 cm^−1^ corresponds to borate, exhibiting the characteristic B–O stretching vibrations in trigonal BO_3_ units.^59–62^ With the addition of SrO, the band shifted towards the lower end, from the ∼1570 cm^−1^ region to the ∼1300 cm^−1^ region. The redshift is due to a weakening of the B–O bond in BO_3_ units, caused by the modifier. By interacting with NBOs, the Sr^2+^ cations might lower the local electron density surrounding boron, which in turn lowers the vibrational frequency of the BO_3_ units. The weak band around 1174 cm^−1^ was due to asymmetrical stretching of the B–O bond in trigonal BO_3_ units.^63^ The intensity of this band increases with the SrO addition and is clearly visible in the KS-35 and KS-40 glasses. The band appearing near ∼1050 cm^−1^ and ∼1033 cm^−1^ corresponds to Q^3^ silicate units.^64^ This region shows no significant changes in the band with the addition of SrO. The band at ∼1004 to 1008 cm^−1^ corresponds to BO_4_ tetrahedra, and there was no obvious change with the addition of SrO. However, this band overlaps with stretching vibrations of SiO_4_, which are mostly attributed to silicate units (Si–O–Si asymmetric stretching in SiO_4_ units), which are made up of two BOs (Q^2^ units).^64,65^ Since the BO_4_ tetrahedron band in this region overlaps with the stretching vibrations of the SiO_4_ tetrahedron, it appears as a small kink. The bands found near ∼897 cm^−1^ and ∼857 cm^−1^ are associated with Si–O^−^ asymmetric stretching vibrations, corresponding to Q^2^ and Q^1^ silicate structural units, respectively.^66^

The intensity of band Q^1^ is highest for the KS-25 glass as compared to the other glasses. With sequential substitution of monovalent K^+^ cations by divalent Sr^2+^ cations, the Q^1^ band intensity decreases, and the Q^2^ band becomes prominent for the KS-35 and KS-40 glasses. Transmission bands located near ∼727–705 cm^−1^ correspond to the Si–O bending and B–O–B bending vibrations.^67^ One report suggests that the band near 690 cm^−1^ probably originates from vibrations of a ring-type metaborate group and the band near 730 cm^−1^ probably originates from vibrations of a chain-type structure.^68^ Thus, the band shift occurring at ∼705 and ∼699 cm^−1^ is related to metaborate bending and shifts toward a higher wavenumber (∼727 and 722 corresponding to the chain-type metaborate group) with an increase in SrO content. These bands at 705–727 cm^−1^ align with the Raman characteristics associated with metaborate groups (663–709 cm^−1^). The 600 to 400 cm^−1^ region corresponds to cation–oxygen bridges, Si–O–Si, B–O–B, O–B–O, and B–O–Si bridging bonds.^19,65,69,70^ This band aligns well with the Raman bands in the 459–486 and 544–554 cm^−1^ regions, assigned to B–O–B, B–O–Si, and Si–O–Si bending vibrations. Thus, the Raman and FTIR spectroscopy data are quite consistent in displaying the structural changes associated with SrO addition in borosilicate glasses.

### 4.7 Optical band gap

The optical band gap gives some idea about the insulating nature of glasses and guides their selection as sealants.^14^ The optical band gap was determined by plotting a graph of (*F*(*R*)*hν*)^2^*versus* energy (*hν*). Here, the Kubelka–Munk function *F*(*R*) is the ratio of the absorption coefficient (*α*) and reflectance for an infinitely thick sample and can be calculated using the following equation:^71^2
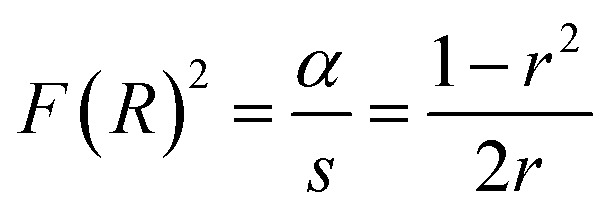
where *r* is reflectance and *s* is the scattering coefficient, respectively.

The optical band gap energy of all the prepared glasses was found to be in the insulating range. It has been reported that when modifiers are introduced in a glass network, it would break the Si–O–Si linkage and create NBOs in the glass network, leading to modification of the optical bands.^72^ As shown in Table 5, the Q^2^ structural units increase at the expense of Q^3^ with increasing SrO content. These defects generate a new localized state across the valence and conduction bands, which in turn reduces the band gap.^73^ Excessive addition of SrO weakens the glass network because weaker bonds easily replace the strong B–O bonds. This weakened bonding in the glass network is the reason for the reduction of the band gap energy. The highest optical band gap of 4.36 eV was obtained for the KS-20 glass, while the lowest band gap of 4.26 eV was observed for the KS-40 glass. The uncertainty in the optical band gap was calculated using the error propagation method from the errors in the slope and intercept obtained from the linear fitting of the Tauc plots. The maximum uncertainty in the optical band gap was ±0.06 eV. In fact, the change in the optical band gap is not very large (∼0.1 eV) with respect to the increase in SrO content in place of K_2_O in the present glasses. But a systematic decrease with composition is identified, and this is consistent with the increase in NBO concentration, molar volume, and structural changes that occur in the glass network. The insulating properties of glasses are not drastically changing with the addition of SrO content. So, these glasses can work as a sealant in SOFCs, with improved fracture toughness as discussed in Section 4.8.

#### 4.7.1 Urbach energy

In order to determine the Urbach energy (*E*_u_) of the prepared borosilicate glass series, the graph of ln *F*(*R*) *vs. hν* is plotted. The reciprocal of the slope of the linear portion was taken, and the equation was used to calculate the Urbach energy^14^ as follows:3
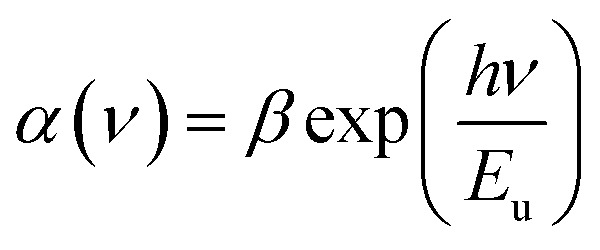


where, *h* is Plank's constant, *ν* is the frequency of light and *β* is the proportionality constant. The Urbach energy of these prepared borosilicate glasses was quite high, at 0.92, 0.87, 0.81, 0.86, and 1.12 for KS-20, KS-25, KS-30, KS-35, and KS-40, respectively. Similar results were reported for Nd-doped borosilicate glass, with Urbach energy values ranging from 1.020 to 1.076 eV for different compositions.^74^ The highest Urbach energy was recorded for the sample KS-40, which exhibits the lowest optical band gap. The above results indicate that the replacement of K_2_O by SrO introduced higher disorder in the present glasses.

#### 4.7.2 Optical basicity

Optical basicity is calculated and correlated qualitatively to understand the structural changes with SrO content. The term “optical basicity” was introduced by Duffy and Ingram for distinguishing the acidic–basic as well as the covalent and ionic nature of glasses. The oxygen ions act as Lewis bases while the metal ions act as Lewis acids. The theoretical optical basicity equation can be represented as:

4 *Λ* = *X*_SiO_2__*Λ*_SiO_2__ + *X*_B_2_O_3__*Λ*_B_2_O_3__ + *X*_K_2_O_*Λ*_K_2_O_ + *X*_SrO_*Λ*_SrO_ + *X*_SnO_2__*Λ*_SnO_2__

where *Λ*_SiO_2__, *Λ*_B_2_O_3__, *Λ*_K_2_O_, *Λ*_SrO_, and *Λ*_SnO_2__ represent the optical basicity values of the constituent oxides. *X*_(SiO_2_)_, *X*_(B_2_O_3_)_, *X*_(K_2_O)_, *X*_(SrO)_, and *X*_(SnO_2_)_ are the equivalent fractions for the different oxides. The values have been taken from the literature for the following oxides: *Λ*_SiO_2__ = 0.50, *Λ*_B_2_O_3__ = 0.425, *Λ*_K_2_O_ = 1.4, *Λ*_SrO_ = 1.14, and *Λ*_SnO_2__ = 0.85.^75,76^ The optical basicity is found to decrease with the increase in SrO content. This is because K_2_O has a higher optical basicity of ∼1.4 compared to that of SrO, which is ∼1.14; as a result, the ability of oxide ions to transfer charge to cations decreased. It may lower the tendency to react with other components of the SOFC. However, it also depends on the chemical nature of the other components of the SOFC and the operating conditions. The reduced basicity will increase the chemical durability with reduced wettability and TEC of the glass sealants. Thus, the glasses containing mixed alkali and alkaline earth oxide, *i.e.*, KS-20 and KS-25, could be a good choice as a sealant for IT/LT-SOFCs.

### 4.8 Mechanical properties

A glass sealant not only prevents the mixing of the fuels and air but also supports the anode-supported SOFC. Thus, the hardness and fracture toughness of a glass sealant are important properties. In particular, a glass sealant must have higher fracture toughness as a sealant. The relation given by Makishima and Mackenzie was used to determine the mechanical strength of the prepared borosilicate glass samples.^77^ This can be described by various mechanical factors, such as the packing density (*V*_t_), dissociation energy per unit volume (*G*_*i*_), and elastic modulus (*E*), as mentioned below:^14,77^5
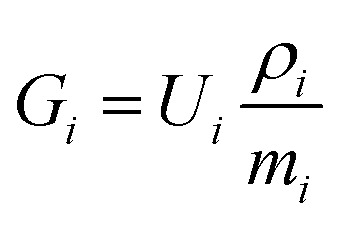
6
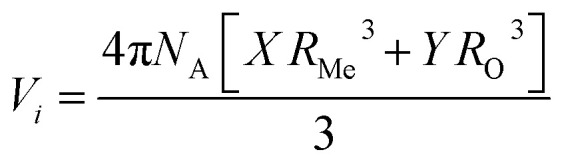
7
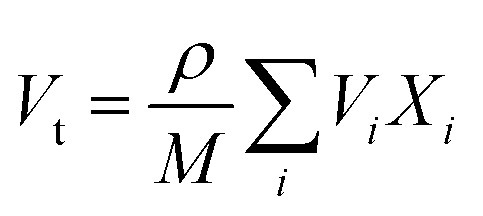
8
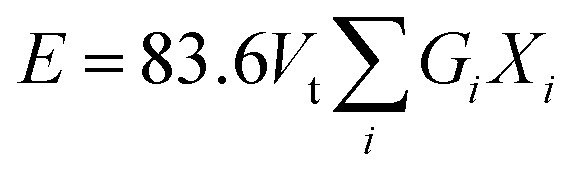
where *m*_*i*_ is the molecular weight, *G*_*i*_ is the dissociation energy per unit volume (kcal cm^−3^), *V*_*i*_ is a packing factor, and *X*_*i*_ is the mole fraction of the *i*th oxide component, respectively. *N*_A_ is Avogadro’s number = 6.022 × 10^23^, and *R*_Me_ and *R*_O_ represent Pauling’s ionic radii of the metal cation and oxygen, respectively. *M* represents the effective molecular weight, and ρ represents the density. The values for different oxides (*U*_*i*_, *G*_*i*_) are taken from the literature, namely SiO_2_ (424, 15.4), B_2_O_3_ (712, 18.6), K_2_O (230, 5.6), SrO (256, 11.6), and SnO_2_ (278, 12.9), respectively.^77^ The *V*_t_ values for KS-20, KS-25, KS-30, KS-35, and KS-40 are 0.62, 0.66, 0.67, 0.66, and 0.64, respectively. The decrease in *V*_t_ for the KS-35 to 40 sample was accompanied by a constant *V*_m_. The value of the elastic modulus increases with increasing SrO content because the Sr^2+^ cation has a higher field strength than the K^+^ cation, except for sample KS-40. This observed trend in the elastic modulus of the glass system was due to the significant effect of replacing one modifier ion with another. In the case of the KS-40 sample, there was a single modifier ion and comparable field strength for KS-35 and KS-40. This might be the reason for a slight decrease in the elastic modulus compared to that of the KS-35 sample. On the other hand, the value of the elastic modulus is lowest when an equal proportion of modifier ions (K_2_O and SrO) is used.

**Fig. 9:**
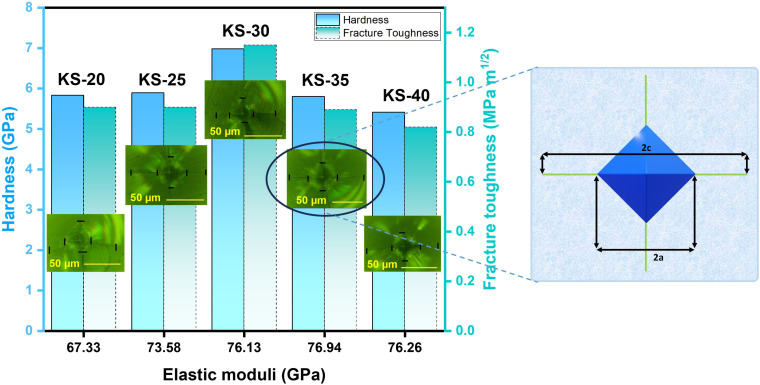
Vicker’s hardness, fracture toughness, and elastic moduli of KS-20, KS-25, KS-30, KS-35, and KS-40 glasses, with representative Vickers indentation images showing a radial/median crack geometry. The inset represents a schematic diagram illustrating the indentation diagonal (2*a*) and crack length (2*c*) used for toughness calculation.

The cracks induced in the borosilicate glass during indentation testing exhibit a median/radial crack system (*c*/*a* ≥ 2.5), as shown in Fig. 9. The hardness value of strontium borosilicate glasses decreases with the increase in SrO content.^32^ The hardness values observed in the present glasses are comparable to those reported for similar types of glass compositions. However, they do not decrease with the SrO content. This can be associated with the combined effect of both the modifiers K_2_O and SrO. The prepared samples do not follow any trend for hardness, firstly increasing from KS-20 to KS-30 with an increase in SrO. This might be due to the presence of two modifiers (alkali and alkaline earth metal). A higher value of hardness corresponds to strong structural bonding within the glass network, leading to greater compactness and higher density of the glasses. Then there is a decrease from KS-35 to KS-40 with an increase in the SrO oxide content. This is because at high concentrations, Sr^2+^ enters into the borosilicate network and replaces the stronger Si–O–Si and B–O–B bonds with Si–O–Sr and B–O–Sr.

The indentation fracture toughness value was first proposed by Anstis *et al.*, who stated the relation between hardness *H*_v_, Young’s modulus *E*, the crack length *c*, and the applied load *F*, as given in eqn (9).^78^ Elsewhere, Lawn and Marshal proposed the method for calculating the brittleness index, as stated in eqn (10).^79^ The results showed that the fracture toughness remains nearly constant for KS-20 and KS-25 samples and increases for the KS-30 sample. The further addition of SrO decreased the fracture toughness value going from KS-35 to KS-40. Thus, the results indicate that a moderate amount of SrO improves resistance to crack propagation, whereas excessive SrO weakens the glass network.9
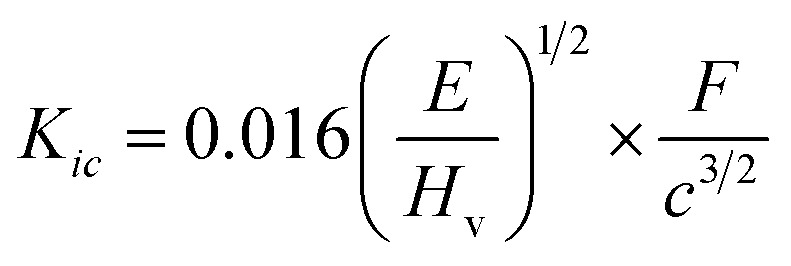
10
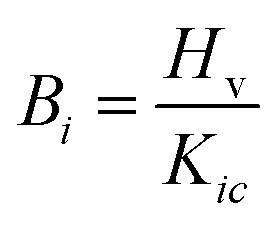


The brittleness indices for the KS-20, KS-25, KS-30, KS-35, and KS-40 samples are 6.47, 6.51, 6.08, 6.51, and 6.61 µm^−1/2^, respectively. The results illustrate that sample KS-30 has the lowest brittleness, followed by KS-20 and KS-25, demonstrating higher mechanical stability than the KS-35 and KS-40 samples. However, the only parameter that indicates whether a glass is a good sealant material for SOFC applications is its mechanical properties. The thermal expansion compatibility with other adjoining components (interconnect, cathode, electrolyte, *etc.*) is also a critical parameter for suitable glass sealants.

### 4.9 Thermal properties

A dilatometric study was performed to investigate the thermal properties of the as-prepared glasses. Various reported studies suggest that for developing effective glass sealant, *T*_g_ should be in the temperature ranges of 650–750 °C/450–650 °C for IT/LT-SOFCs, respectively. Similarly, the *T*_s_ value must be higher than the cell operating temperature to prevent excessive viscous flow during operation. The ranges of *T*_s_ should be 700–900 °C/500–700 °C for IT/LT-SOFCs, respectively.^12^ Moreover, TEC is a crucial parameter to ensure thermal suitability of glass sealants with other components of SOFCs. Generally, the TEC of glasses lies in the range of 9 to 13 × 10^−6^ °C^−1^, and can be used as a sealant for SOFCs.^12^ The *T*_s_ values of the present glasses were found to be 662 °C, 708 °C, 776 °C, 745 °C, and 704 °C for KS-20, KS-25, KS-30, KS-35, and KS-40, respectively. All these glasses fall within the ranges for IT/LT-SOFCs, confirming their suitability to be used as glass sealants. Additionally, the TECs of the glass samples vary non-linearly, and lie in the range of 12 to 18 × 10^−6^ °C^−1^ for KS-20 to KS-40. This TEC value was taken in the range of 400 to 600 °C. The KS-20 and KS-25 glasses exhibit TEC values around 13 × 10^−6^ °C^−1^ and 12 × 10^−6^ °C^−1^, respectively. Meanwhile, the TECs of the KS-30, KS-35, and KS-40 samples are around 17 × 10^−6^ °C^−1^, 18 × 10^−6^ °C^−1^, and 16 × 10^−6^ °C^−1^, respectively.

The observed changes in the *T*_s_ value within the SiO_2_–B_2_O_3_–SnO_2_ glass network were attributed to the structural modifications caused by the substitution of the monovalent K^+^ cation by the divalent Sr^2+^ cation. The incorporation of alkali and alkaline earth modifier oxides promotes the formation of NBOs. However, Sr^2+^ exhibits greater field strength compared to K^+^, resulting in an increase in bond strength. This partially enhanced the rigidity of the glass network, leading to an increase in *T*_s_ at intermediate compositions. This result aligns well with previous studies, which suggest an increase in *T*_s_ and TEC with the addition of SrO content.^11^ A comparison of the *T*_s_ and TEC properties for various borosilicate glass compositions from the literature and the present study is provided in Table 6. An earlier report also indicated that SnO_2_ addition up to 0.5 wt% lowered the TEC, and above 0.5 wt%, an increase in TEC was observed.^19^ The reason for such a high TEC above 30 mol% SrO content might the combined effect of excessive SrO modifier and the effect of SnO_2_, which weakened the structure and increased the TEC. This happened because of the creation of more NBOs in the glass network, which breaks Si–O–Si and B–O–B bonds, which are replaced by Si–O–Sr and B–O–Sr, as stated earlier in the Raman spectral analysis. Weaker Sr–O bonds result in more thermal expansion due to the asymmetric nature of the potential energy *versus* inter-atomic distance. However, the TEC value of KS-40 is lower than that of the KS-35 sample, which might be due to the presence of a single modifier. Despite this, weaker bonds at higher SrO content decreased the network connectivity and consequently lowered the *T*_s_.

**Table 6:** Comparison of *T*_s_ and TEC of borosilicate glass compositions from the literature and this work

Glass composition	*T*_s_ (°C)	TEC (×10^−6^ °C^−1^)	Ref.
35AO–50B_2_O_3_–15SiO_2_, (A = Ba, Ca, Sr)	—	8.18–10.15	80
20SrO–20BaO–*x*B_2_O_3_–(60 − *x*)SiO_2_, 10 ≤ *x* ≤ 40	702–660	9.5–7.5	16
15La_2_O_3_–15Al_2_O_3_–30SiO_2_–*x*SrO–(40 − *x*)B_2_O_3_, 10 ≤ *x* ≤ 30	660–709	8.29–9.72	11
(40 − *x*)SrO–*x*BaO–45SiO_2_–10B_2_O_3_–5ZrO_2_, 0 ≤ *x* ≤ 40	571–730	7.75–14	14
17BaO–17CaO–5Al_2_O_3_–*x*B_2_O_3_–(61 − *x*)SiO_2_, 0 ≤ *x* ≤ 9	844–945	10.0–11.0	3
40SiO_2_–15B_2_O_3_–(40 − *x*)K_2_O–*x*SrO–5SnO_2_, 20 ≤ *x* ≤ 40	662–776	12–18	This work

Cr-based alloys and a metallic interconnect based on Fe stainless steel could be a suitable choice for KS-20 and KS-25 samples due to the TEC value falling within the range of 11–14 × 10^−6^ °C^−1^.^81^ Such close matching might show good thermal and mechanical compatibility between the developed glasses and typical SOFC interconnect materials, which is essential for reliable sealing at elevated temperatures. On the other hand, the KS-30, KS-35, and KS-40 samples might be compatible with Ni-based superalloys, which exhibit TEC values in the range of 14–19 × 10^−6^ °C^−1^.^81^ But considering other components, such as YSZ or Ceria-based electrolytes that exhibit TECs around 11–12 × 10^−6^ °C^−1^, the TEC mismatch becomes very large and might cause leakage.^82,83^ One of the previous studies suggests that borosilicate glass containing SrO/BaO modifier exhibits strong interfacial adhesion between YSZ and the glass.^84^ Therefore, based upon all glasses, the KS-20 and KS-25 glasses might show good compatibility with other components for SOFC applications.

## 5 Conclusion

The density and phase separation of SnO_2_-containing borosilicate glasses increase as SrO is replaced by K_2_O. However, the density becomes constant at higher SrO concentrations, particularly in the KS-35 and KS-40 samples. The addition of SrO also facilitates higher phase separation by raising the concentration of NBOs. Within the glass network, tin is mainly present in two oxidation states: Sn^2+^ and Sn^4+^. The optical band gap energy decreases marginally with SrO addition but remains within the insulating range (4.36 to 4.26 eV). The synthesized glasses exhibit robust mechanical properties, including high hardness, Young’s modulus, and fracture toughness, with the highest fracture toughness (∼1.15) observed in the KS-30 sample. The mechanical characteristics did not show a linear trend with the addition of SrO content. However, TEC also varies non-linearly with the addition of SrO content. For glass samples KS-30 to KS-40, the TEC values (16 to 18 × 10^−6^ °C^−1^) are higher than required for SOFC applications. Based on the results, it is found that KS-20 and KS-25 could be suitable sealant materials due to their favorable fracture toughness, hardness, and thermal compatibility for IT/LT SOFCs. However, long-term chemical compatibility and stability under reducing and humidified hydrogen atmospheres require further investigation for these developed glasses.

## Conflicts of interest

No conflicts of interest exist to disclose.

## Supplementary Material

RA-016-D5RA10040B-s001

## Data Availability

Data will be made available on request. Supplementary information (SI) is available. See DOI: https://doi.org/10.1039/d5ra10040b.
