# Supplementary material for: Effect of K2O/SrO on structural, thermal, optical, and mechanical properties of SiO2–B2O3–SnO2 glass for IT/LT-SOFC applications
Source: RSC Adv. 2026 May 18;16(28):26083–98. doi: 10.1039/d5ra10040b (PMC13185698; doi:10.1039/d5ra10040b)
Supplement: RA-016-D5RA10040B-s001 [file RA-016-D5RA10040B-s001.pdf]

Supplementary Information (SI) for RSC Advances.
This journal is © The Royal Society of Chemistry 2026

K_2O/SrO effect on structural, thermal, optical, and mechanical properties of SiO_2-B_2O_3-SnO_2 glass for IT/LT-SOFC application

Manish Kumar[1,2], Akshay Kumar[1], K. Singh[2,*]

[*]Corresponding author: kusingh@thapar.edu
[1]Department of Physics, Sardar Patel University, Mandi, Himachal Pradesh-175001, India
[2]Department of Physics and Material Science, Thapar Institute of Engineering and Technology, Patiala-147004, Punjab, India

Electronic Supplementary Information

1 Raman analysis

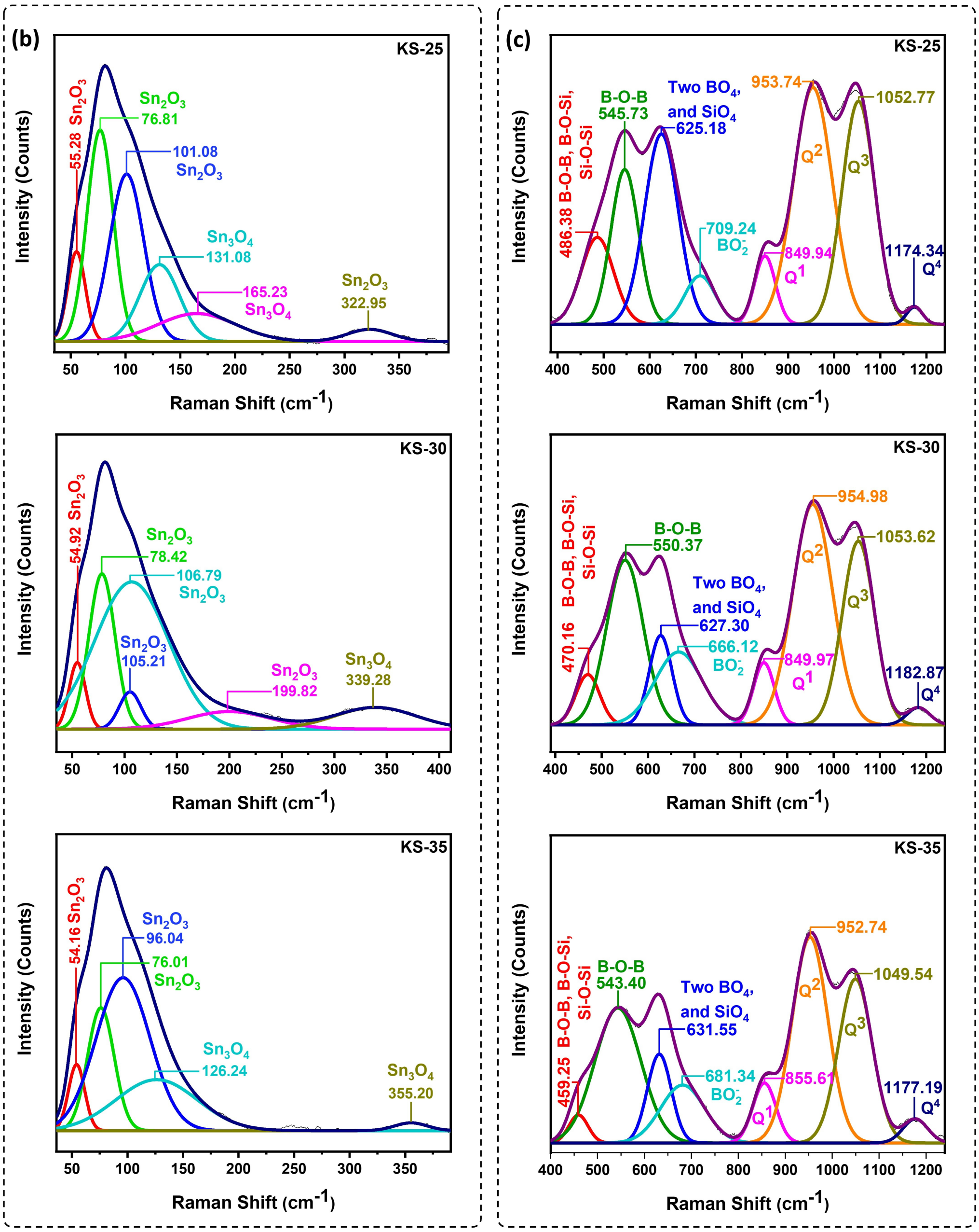

Figure S 1: (a) Representative deconvoluted spectra of KS-25, KS-30, and KS-35 glasses in the spectral range of below 400 cm^{-1}, and (c) Representative deconvoluted spectra of KS-25, KS-30 and KS-35 glasses in the range of 400-1200 cm^{-1}.

1.1 Raman peak fitting parameters

- **KS-20**

Table S 1: Raman peak fitting parameters for normalized spectrum below 400 cm^{-1}.

	y_0		x_c		A		w		Statistics		
Peak	Value	Standard Error	Value	Standard Error	Value	Standard Error	Value	Standard Error	Red. χ^2	Adj. R^2	Residual Sum of squares
Peak1	0.02307	4.12E-04	56.1706	0.21239	4.41058	0.37821	17.76551	0.40042	1.68E-05	0.99971	0.01177
Peak2	0.02307	4.12E-04	77.15995	0.66249	17.81908	2.03991	27.81399	1.13929			
Peak3	0.02307	4.12E-04	99.69558	2.15868	11.851	5.1062	33.87486	4.10286			
Peak4	0.02307	4.12E-04	121.6121	3.92455	22.21909	4.4318	57.39228	4.52613			
Peak5	0.02307	4.12E-04	184.1234	3.17008	8.31194	0.66929	89.02201	3.6102			
Peak6	0.02307	4.12E-04	316.249	0.48161	1.08737	0.0421	38.1865	1.31647			

Table S 2: Raman peak fitting parameters for the normalized spectrum in the region 400 to 1200 cm^{-1} of KS-20 sample

	y_0		x_c		A		w		Statistics		
Peak	Value	Standard Error	Value	Standard Error	Value	Standard Error	Value	Standard Error	Red. χ^2	Adj. R^2	Residual Sum of squares
Peak1	0.01498	8.17E-04	473.46994	2.57442	14.2138	2.5422	68.859	2.55501	9.52E-05	0.99914	0.17725
Peak2	0.01498	8.17E-04	543.10447	0.88918	69.05922	3.81599	94.11653	4.36602			
Peak3	0.01498	8.17E-04	619.82552	0.62442	27.56967	3.213	61.76788	1.79275			
Peak4	0.01498	8.17E-04	663.00627	3.66762	38.39473	3.06176	108.54937	3.60519			
Peak5	0.01498	8.17E-04	849.04791	0.20016	11.17296	0.14549	49.14865	0.49361			
Peak6	0.01498	8.17E-04	958.32898	0.1767	107.25101	0.49187	109.43896	0.48502			
Peak7	0.01498	8.17E-04	1057.3216	0.1445	76.63474	0.42404	79.89771	0.24314			
Peak8	0.01498	8.17E-04	1173.5	0.77058	2.16777	0.10606	47.90327	2.09955			

- **KS-25**

Table S 3: Raman peak fitting parameters for normalized spectrum below 400 cm^{-1}.

	y_0		x_c		A		w		Statistics		
Peak	Value	Standard Error	Value	Standard Error	Value	Standard Error	Value	Standard Error	Red. χ^2	Adj. R^2	Residual Sum of squares
Peak1	0.03736	6.48E-04	55.28783	0.2194	6.35979	0.36411	19.179	0.2741	1.99E-05	0.99975	0.01364
Peak2	0.03736	6.48E-04	76.81196	0.57277	21.98171	3.23781	28.16322	1.11094			
Peak3	0.03736	6.48E-04	101.0896	0.90634	22.60812	5.88725	36.59315	4.25982			
Peak4	0.03736	6.48E-04	131.0857	4.19492	12.03244	4.10072	42.39549	4.57406			
Peak5	0.03736	6.48E-04	165.2373	5.65785	8.70302	1.38792	84.30044	5.81412			
Peak6	0.03736	6.48E-04	322.9591	0.36429	2.17661	0.07488	48.41467	1.26355			

Table S 4: Raman peak fitting parameters for the normalized spectrum in the region 400 to 1200 cm^{-1} of KS-25 sample

	y_0		x_c		A		w		Statistics		
Peak	Value	Standard Error	Value	Standard Error	Value	Standard Error	Value	Standard Error	Red. χ^2	Adj. R^2	Residual Sum of squares
Peak1	0.00615	0.0011	486.3835	2.80802	29.11756	2.49986	83.29069	2.75207	1.19E-04	0.99876	0.21639
Peak2	0.00615	0.0011	545.7312	0.81341	44.15797	3.54371	70.89888	2.22203			
Peak3	0.00615	0.0011	625.183	0.48769	65.70438	2.27019	85.78323	2.26669			
Peak4	0.00615	0.0011	709.2455	1.99284	15.30088	1.03715	78.4675	2.40195			
Peak5	0.00615	0.0011	849.9377	0.21145	14.59768	0.18851	53.10784	0.51348			
Peak6	0.00615	0.0011	953.7395	0.18698	95.0567	0.5232	99.72849	0.52444			
Peak7	0.00615	0.0011	1052.774	0.19262	75.54236	0.48214	84.16229	0.32732			
Peak8	0.00615	0.0011	1174.34	0.50725	2.80202	0.11436	40.39047	1.42357			

- **KS-30**

Table S 5: Raman peak fitting parameters for normalized spectrum below 400 cm^{-1}.

	y_0		x_c		A		w		Statistics		
Peak	Value	Standard Error	Value	Standard Error	Value	Standard Error	Value	Standard Error	Red. χ^2	Adj. R^2	Residual Sum of squares
Peak1	-0.0133	0.0038	54.92367	0.21666	4.94867	0.38873	18.50264	0.50648	2.96E-05	0.99964	0.02125
Peak2	-0.0133	0.0038	78.42958	0.12667	18.33356	0.56964	29.348	0.8612			
Peak3	-0.0133	0.0038	105.2179	0.82869	3.62118	0.54111	24.22341	1.2925			
Peak4	-0.0133	0.0038	106.7924	0.70507	48.81885	1.08989	82.46893	1.11368			
Peak5	-0.0133	0.0038	199.8253	3.13446	6.62593	0.60459	93.75928	5.11281			
Peak6	-0.0133	0.0038	339.2806	0.493	7.93756	0.63281	91.00105	3.48381			

Table S 6: Raman peak fitting parameters for the normalized spectrum in the region 400 to 1200 cm^{-1} of KS-30 sample

	y_0		x_c		A		w		Statistics		
Peak	Value	Standard Error	Value	Standard Error	Value	Standard Error	Value	Standard Error	Red. χ^2	Adj. R^2	Residual Sum of squares
Peak1	0.00272	9.89E-04	470.1634	0.60121	12.3009	0.49216	60.82341	0.98816	5.31E-05	0.99928	0.09587
Peak2	0.00272	9.89E-04	550.374	0.43372	61.20713	1.19271	92.38942	1.3609			
Peak3	0.00272	9.89E-04	627.2995	0.29809	20.71865	1.37321	57.63732	1.05133			
Peak4	0.00272	9.89E-04	666.1207	3.17297	35.25029	2.18696	120.214	3.44887			
Peak5	0.00272	9.89E-04	849.9686	0.15325	13.23629	0.13061	52.89882	0.38023			
Peak6	0.00272	9.89E-04	954.9805	0.13351	89.58509	0.3569	100.9649	0.37719			
Peak7	0.00272	9.89E-04	1053.616	0.14932	60.72508	0.3237	82.16973	0.26019			
Peak8	0.00272	9.89E-04	1182.872	0.41374	4.02402	0.12736	58.05731	1.34104			

- **KS-35**

Table S 7: Raman peak fitting parameters for normalized spectrum below 400 cm^{-1}.

Peak	y_0 Value	y_0 Standard Error	x_c Value	x_c Standard Error	A Value	A Standard Error	w Value	w Standard Error	Statistics Red. χ^2	Statistics Adj. R^2	Statistics Residual Sum of squares
Peak1	0.16595	5.45E-04	54.16724	0.20574	3.83783	0.33728	17.31208	0.53889	4.88E-05	0.9992	0.03478
Peak2	0.16595	5.45E-04	76.01835	0.23301	11.61747	1.76981	28.19476	0.96965			
Peak3	0.16595	5.45E-04	96.04014	1.78975	29.33584	13.34054	57.17887	6.72935			
Peak4	0.16595	5.45E-04	126.2496	24.55725	14.73698	11.65213	86.17484	16.57519			
Peak5	0.16595	5.45E-04	355.2037	0.8213	0.90557	0.06104	34.6487	2.216			

Table S 8: Raman peak fitting parameters for the normalized spectrum in the region 400 to 1200 cm^{-1} of KS-35 sample

Peak	y_0 Value	y_0 Standard Error	x_c Value	x_c Standard Error	A Value	A Standard Error	w Value	w Standard Error	Statistics Red. χ^2	Statistics Adj. R^2	Statistics Residual Sum of squares
Peak1	0.1309	0.00106	459.2497	0.4033	4.51935	0.27649	48.22967	1.34986	3.94E-05	0.99894	0.06946
Peak2	0.1309	0.00106	543.4008	0.30025	52.33016	0.66419	115.7224	1.50686			
Peak3	0.1309	0.00106	631.5511	0.25958	17.89265	1.09643	60.10321	1.03479			
Peak4	0.1309	0.00106	681.3455	2.81326	21.32343	1.314	109.9561	3.47624			
Peak5	0.1309	0.00106	855.6108	0.20191	11.65388	0.14341	57.9896	0.4902			
Peak6	0.1309	0.00106	952.7412	0.12544	61.79815	0.29674	89.55689	0.39347			
Peak7	0.1309	0.00106	1049.542	0.17578	46.25663	0.28174	84.14141	0.33894			
Peak8	0.1309	0.00106	1177.189	0.33256	4.97266	0.13494	62.00259	1.12996			

- **KS-40**

Table S 9: Raman peak fitting parameters for normalized spectrum below 400 cm^{-1}.

Peak	y_0 Value	y_0 Standard Error	x_c Value	x_c Standard Error	A Value	A Standard Error	w Value	w Standard Error	Statistics Red. χ^2	Statistics Adj. R^2	Statistics Residual Sum of squares
Peak1	0.11559	5.18E-04	53.09204	0.14698	2.27889	0.16633	14.77648	0.48918	3.87E-05	0.99934	0.02559
Peak2	0.11559	5.18E-04	76.57989	0.22582	16.14297	1.44677	35.22637	0.69234			
Peak3	0.11559	5.18E-04	97.18416	1.88072	29.11423	9.77642	66.59474	6.117			
Peak4	0.11559	5.18E-04	142.8708	26.54847	15.5234	8.8912	113.2327	27.31031			
Peak5	0.11559	5.18E-04	247.0995	0.68069	1.12306	0.1889	33.84891	2.86318			

Table S 10: Raman peak fitting parameters for the normalized spectrum in the region 400 to 1200 cm^{-1} of KS-40 sample

Peak	y_0		x_c		A		w		Statistics		
	Value	Standard Error	Value	Standard Error	Value	Standard Error	Value	Standard Error	Red. χ^2	Adj. R^2	Residual Sum of squares
Peak1	0.08104	0.00113	475.0307	0.91745	12.79184	0.49961	71.85398	1.44767	3.08E-05	0.99812	0.05388
Peak2	0.08104	0.00113	548.6886	0.47952	23.56274	0.61197	75.74466	1.43034			
Peak3	0.08104	0.00113	629.3268	0.30405	14.22381	0.85965	61.04318	1.17219			
Peak4	0.08104	0.00113	682.5886	2.74052	27.21731	1.24826	139.143	3.79569			
Peak5	0.08104	0.00113	847.9626	0.28348	7.76866	0.14706	59.93628	0.73801			
Peak6	0.08104	0.00113	955.4691	0.12407	55.31858	0.29314	111.5095	0.55931			
Peak7	0.08104	0.00113	1055.61	0.14992	22.0821	0.19117	66.66812	0.31954			
Peak8	0.08104	0.00113	1167.361	0.23618	5.47626	0.13665	58.55284	0.93107			

2 FTIR analysis

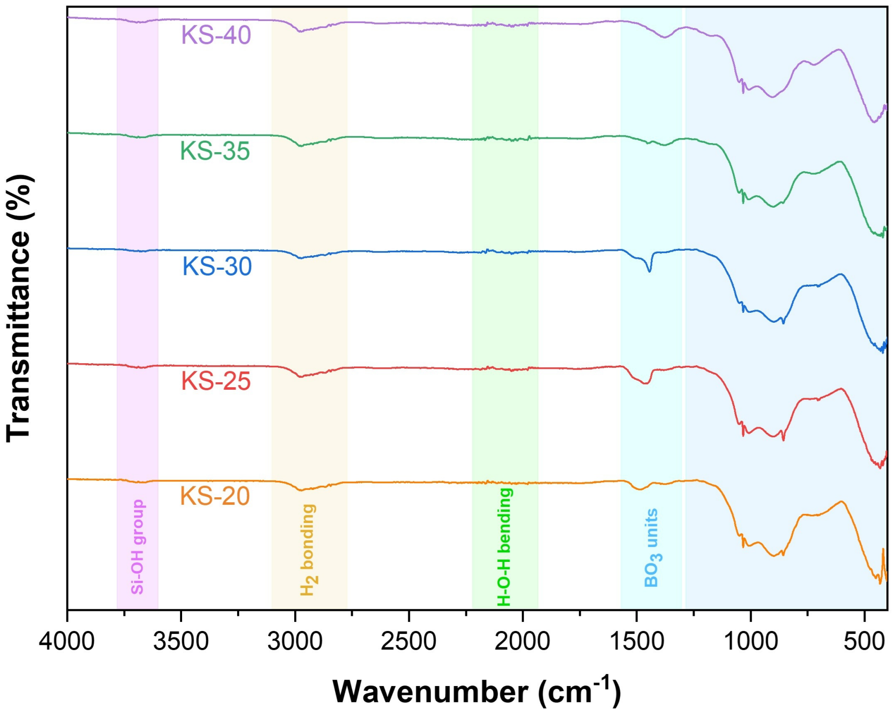

Figure S 2: FTIR transmittance spectra of the prepared glass series over the full wavenumber range (4000–400 cm^{-1}).
